# Enhanced Mask R-CNN Incorporating CBAM and Soft-NMS for Identification and Monitoring of Offshore Aquaculture Areas

**DOI:** 10.3390/s25092792

**Published:** 2025-04-29

**Authors:** Jiajun Zhang, Yonggui Wang, Yaxin Zhang, Yanxin Zhao

**Affiliations:** 1 Hubei Key Laboratory of Regional Ecology and Environmental Change, School of Geography and Information Engineering, China University of Geosciences, Wuhan 430079, China; jiajunz@cug.edu.cn (J.Z.); wangyg@cug.edu.cn (Y.W.); 1202021066@cug.edu.cn (Y.Z.); 2 State Key Laboratory of Hydraulic Engineering Intelligent Construction and Operation, Tianjin University, Tianjin 300072, China; 3 Yellow River Ecology and Environment Protection Center, Chinese Academy of Environmental Planning, Beijing 100041, China

**Keywords:** offshore aquaculture, Mask R-CNN, trend analysis, violation monitoring, CBAM and Soft-NMS

## Abstract

The use of remote sensing images to analyze the change characteristics of large-scale aquaculture areas and monitor aquaculture violations is of great significance for exploring the law of marine aquaculture and assisting the monitoring and standardization of aquaculture areas. In this study, a violation monitoring framework for marine aquaculture areas based on image recognition using an enhanced Mask R-CNN architecture incorporating a convolutional block attention module (CBAM) and soft non-maximum suppression (Soft-NMS) is proposed and applied in Sandu’ao. The results show that the modified Mask R-CNN, when compared to the most basic Mask R-CNN model, exhibits higher accuracy in identifying marine aquaculture areas. The aquaculture patterns in the Xiapu region are characterized by two peak periods of aquaculture area fluctuations, occurring in March and October. Conversely, July marks the month with the smallest aquaculture area in the region and is influenced by factors such as water temperature and aquaculture cycle. Significant changes in the aquaculture area were observed in January, March, June, August, and October, necessitating rigorous monitoring. Furthermore, monitoring and analysis of aquaculture areas have revealed that despite the reduction in illegal aquaculture acreage since 2017 due to the implementation of functional zone planning for marine aquaculture areas, illegal aquaculture activities remain prevalent in prohibited and restricted zones in Xiapu, accounting for a considerable proportion.

## 1. Introduction

In recent years, over-cultivation in mariculture areas has led to increasingly severe marine environmental pollution. Countries such as New Zealand [1,2,3] have faced significant challenges related to excessive aquaculture pollution. Management strategies have gradually shifted from prohibiting marine aquaculture to a more nuanced approach, which involves reasonable planning of marine aquaculture areas and allowing for a certain scale of over-cultivation in specific areas and periods. Therefore, accurately analyzing local aquaculture behavior to determine regional aquaculture change trends is essential. Identifying the main time and areas where aquaculture behavior changes during the process is crucial for conducting key monitoring. Monitoring violations in key areas of marine aquaculture can provide early warnings for over-cultivation, assist management departments in dealing with illegal aquaculture promptly, and more effectively prevent marine pollution caused by over-cultivation.

Exploring the evolutionary trend of aquaculture and determining the key points of violation monitoring require accurate and long-term data analysis. However, due to the location of aquaculture areas in the ocean, arranging detection instruments is challenging due to factors such as waves. Long-term fixed-point manual observation and data collection, which are relatively easier on land, are difficult to implement in marine environments. As a result, most marine aquaculture areas lack historical monitoring data and, even more so, large-scale overall monitoring data. Remote sensing technology has developed rapidly, and its application for regional status monitoring has become widespread [4,5]. Some research has also used remote sensing to analyze regional long-term change trends and has achieved good results [6,7]. In recent years, remote sensing monitoring of marine aquaculture areas has made some progress. This progress is mainly reflected in the use of remote sensing images to quickly identify, classify, and analyze aquaculture areas in a short period of time or at a single time point [8,9,10]. Additionally, some scholars have analyzed the changes in aquaculture areas over long time ranges [11,12,13,14,15,16]. However, these studies only reflect overall changes in aquaculture areas over long periods by analyzing changes on a yearly basis. They lack detailed analysis of the aquaculture trend. Moreover, few studies have fully utilized the characteristics of remote sensing to monitor violations in marine aquaculture areas. Given the current need for more refined and quantified management of marine aquaculture areas, monitoring these areas through remote sensing is highly valuable and meaningful. The focus should be on conducting high-precision monitoring of marine aquaculture areas to meet management requirements.

While AI-powered identification models have demonstrated promising results in marine aquaculture monitoring, persistent challenges in land misdetection hinder their operational practicality. A critical limitation arises from the spectral similarity between nearshore aquaculture structures (e.g., raft anchors) and terrestrial features (e.g., harbors, coastal vegetation), leading to significant false positive rates in coastal transition zones [17]. Current mitigation strategies, such as manual NDWI (Normalized Difference Water Index) thresholding coupled with Mask R-CNN, achieve high boundary accuracy but require laborious parameter tuning for different tidal conditions and coastal geometries—a process demanding both domain expertise and computational resources, thus impeding real-time deployment. The evolution of intelligent monitoring tools is now addressing these limitations. For example, a building upon red tide detection framework [18], next-generation models integrate multi-temporal SAR data with optical imagery to distinguish floating aquaculture structures from static land features through dynamic backscatter analysis. Additionally, lightweight model variants enable onboard processing for UAV-based monitoring systems, circumventing cloud dependency while maintaining detection accuracy through neural architecture search (NAS)-optimized networks. Our prior research [17] established that hybrid spectral–deep learning methods can achieve F1 scores of more than 0.90 for RCA/CCA differentiation under optimal conditions. However, practical implementation requires addressing the spectral ambiguity cascade—where cumulative errors from atmospheric correction, water column attenuation, and substrate reflectance interactively amplify land misclassification risks. Herein lies the critical value of integrating a convolutional block attention module (CBAM) and soft non-maximum suppression (Soft-NMS): the CBAM module enhances Mask R-CNN’s feature discrimination capability by adaptively weighting spectral–spatial patterns, effectively isolating aquaculture structures from spectrally similar coastal landscapes (e.g., distinguishing submerged raft anchors from intertidal rocks), while Soft-NMS resolves dense target overlaps through confidence decay rather than binary suppression, preserving valid detections in complex aquaculture clusters [19,20,21]. These advancements fundamentally improve remote sensing interpretation by addressing both pixel-level ambiguity and object-level uncertainty.

This study develops an enhanced Mask R-CNN architecture that innovatively incorporates the convolutional block attention module (CBAM) and soft non-maximum suppression (Soft-NMS), addressing two critical challenges in marine aquaculture monitoring: (1) CBAM-driven adaptive spatial-channel feature weighting enhances discriminative representation of submerged infrastructure in turbid coastal waters, while (2) Soft-NMS resolves overlapping detection artifacts in high-density cultivation zones through probabilistic confidence attenuation, achieving structural compatibility with multispectral remote sensing workflows without requiring architectural modifications. This dual enhancement enables precise delineation of illegal expansion patterns in offshore aquaculture areas while maintaining structural compatibility with standard remote sensing pipelines, establishing a new paradigm for operational marine spatial compliance monitoring.

## 2. Materials and Methods

### 2.1. Study Area and Data Sources

(1) Study area

**Figure 1:**
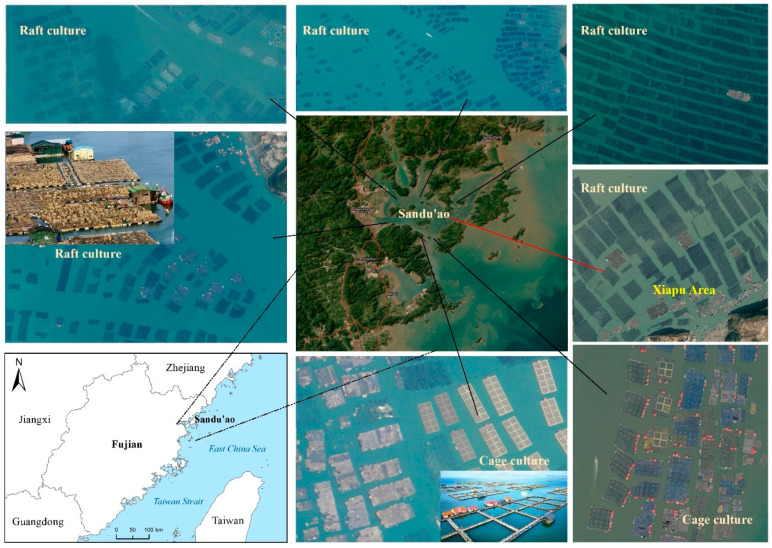
The location and culture areas of the study area (Red line points to Xiapu Area).

Sandu’ao, also known as Sansha Bay, is located in the southeastern part of Ningde City, Fujian Province, China. Covering a sea area of 102 km^2^ with 33 harbor mouths and 16 islands, it offers 110,000 mu (approximately 7333 hectares) of aquaculture space. This area is renowned as the hometown of the large yellow croaker in China, and is a famous fishing ground in eastern Fujian. Within this vast aquaculture zone, 15,000 cage aquaculture farms stretch for dozens of nautical miles, while over 6000 wooden huts bob up and down with waves. Additionally, raft aquaculture areas are scattered throughout and are mainly used for cultivating algae such as dragon’s beard seaweed. The distribution of these farms showcases a harmonious coexistence with the marine environment, ensuring sustainable development of the aquaculture industry, as shown in Figure 1. The lack of real-time monitoring of local aquaculture areas often leads to issues such as over-cultivation and encroachment of non-farming zones, ultimately causing significant marine pollution [17]. Consequently, to align with the government’s mandates for marine ecological conservation, fishery development planning, and the establishment of a modern fishing port system characterized by an aesthetically pleasing environment and orderly management, it is imperative to implement dynamic monitoring of these areas.

(2) Data sources

Remote sensing image data from GF-1 and Landsat-8 were used in this study, as listed in Table 1.

The GF-1 satellite imagery used for model training and validation is in the .tif format. It was acquired on 13 June 2020. The Landsat-8 satellite imagery used is in the .tif format, covering the time period from 2013 to 2021. GF-1 data were used for model training and validation, and Landsat-8 data were used to monitor the changes in the aquaculture area.

During the model training and validation stages, experiments were conducted utilizing remote sensing image data from the Sandu’ao mariculture area, where the dataset was divided into an 80% training set and a 20% verification set.

In the monitoring stage of the aquaculture area, Landsat-8 remote sensing images were used to monitor the changes in aquaculture area in Xiapu (119°49′13″–119°57′3″ E, 26°36′38″–26°41′34″ N) of the Sandu’ao area. The revisit period of the satellite was 16 days, and the spatial resolution was 30 m. After image fusion, the spatial resolution was set to 15 m. According to the coverage of Landsat-8 satellite orbits, all images of different paths and row numbers in this area from 2013 to 2021 were screened from the geospatial data cloud platform (http://www.gscloud.cn/). Finally, 36 high-quality images were obtained for monitoring and analysis, which were not significantly obscured by sediment and clouds.

### 2.2. Method

**Figure 2:**
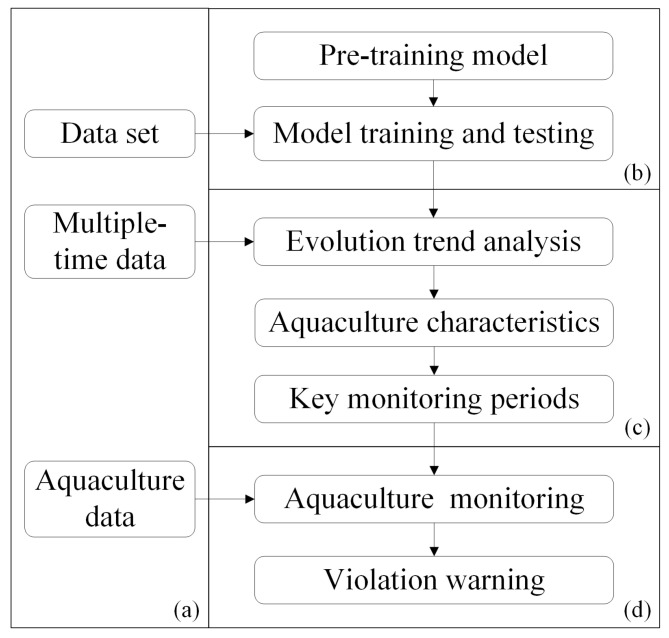
The framework of combining the NDWI and Mask R-CNN for aquaculture areas monitoring. (**a**) data acquisition and pre-processing, (**b**) model construction and verification, (**c**) aquaculture trend analysis, (**d**) violation monitoring.

Based on the identification method combining the NDWI and modified Mask R-CNN, monitoring of aquaculture areas was carried out. The technology framework is illustrated in Figure 2.

As shown in Figure 2, the technical method adopted in this study consists of four steps, as follows:

(a) Data acquisition and pre-processing involves obtaining a dataset of model training and testing, multi-temporal data, and data of aquaculture areas to be monitored.

(b) Model construction and verification are performed by using precision, recall, and *F1* score with the following formulas:

$$Precision=\frac{TP}{TP+FP}$$

$$Recall=\frac{TP}{TP+FN}$$

$$F1\;score=\frac{2\times Precision\times Recall}{Precision+Recall}$$

where *TP* denotes the number of correctly identified pixels in the ground truth, *FP* denotes the number of misidentified pixels that do not exist in the ground truth, and *FN* denotes the number of undetected pixels in the ground truth.

(c) Aquaculture trend analysis involves analyzing the multi-temporal data to obtain the monthly change trend of aquaculture areas and determining the key monitoring period with large changes.

(d) Violation monitoring of aquaculture areas involves inputting the collected key period data into the model, and achieving violation monitoring and warning of prohibited and restricted aquaculture areas by spatial correlation analysis.

#### 2.2.1. Data Pre-Processing

The GF-1 remote sensing images were segmented into 80 samples, each with a size of 500 × 500 pixels. Of these, 64 samples were allocated for training, whereas the remaining 16 were reserved for testing. The boundaries of the raft culture area (RCA) and cage culture area (CCA) within the samples were precisely delineated through manual visual interpretation, resulting in the acquisition of ground-truth data. In this ground truth, the colored blocks represent aquaculture areas, whereas the black blocks indicate the background. Given that the GF-1 images were 10-bit depth, 4-band images were converted into 8-bit RGB images to be compatible with the network input.

Annotation tools included GIS software (ArcMap 10.2) and image editors (GIMP) used on color-calibrated monitors. Color codes were assigned as red (#FF0000) for the RCA, green (#00FF00) for the CCA, and black (#000000) for the background, with boundary rules specifying a minimum unit size of 5 × 5 pixels and ±2-pixel buffer zones for edge tolerance. The annotation process involved dual-blind vectorization by junior annotators and cross-verification by senior interpreters, with feature identification based on texture analysis and spectral validation using the original multispectral data. A three-tier review system ensured high-quality annotations, with a Kappa coefficient ≥ 0.85 and boundary errors ≤ 1.5 pixels. Annotations were stored as vector files (shp and geojson) and raster masks, organized in a structured directory with metadata including annotator IDs, timestamps, and solar elevation angles. The final outputs were converted to single-channel index maps and pseudo-color RGB images for deep learning compatibility. This workflow resulted in high-precision ground-truth data with clear visual and spectral differentiation of aquaculture zones, optimized for training AI models in marine aquaculture monitoring.

Owing to the limited size of the dataset, data augmentation techniques were employed to mitigate overfitting during network training, enhance the robustness of the classifier to variations in sensors, atmospheric conditions, and lighting, and improve the generalization capability of the model. The 64 training images were expanded to simulate diverse image states by applying Gaussian noise, Gaussian blur, and contrast adjustments. This process increased the number of training samples from 64 to 256, and the corresponding ground truth for these augmented samples was also generated.

In addition, in order to verify the recognition effect of the model on remote sensing images with different resolutions, bilinear down-sampling was used to simulate low-resolution images of 4 m, 10 m, 15 m, 20 m, 30 m, and 50 m, respectively, based on the 2 m dataset and the spatial resolution of satellite images commonly used in such studies. The ground truth corresponding to the sample was generated, and datasets with different resolutions were obtained. After pre-processing, the situation of the dataset used for model training and validation is shown in Table 2.

#### 2.2.2. Model Development

(1) Water area extracting model based on the NDWI

For the problem of land misdetection, the *V_NDWI_* was used for water extraction. It is defined as follows:

$$V_{NDWI}=\frac{Green-NIR}{Green+NIR}$$

where *Green* is the green band and *NIR* is the near-infrared band. We selected the appropriate classification threshold to segment the *V_NDWI_*, and then the water area was obtained, thus preventing land misdetection in aquaculture area extraction.

Model development incorporated a spectral analysis framework where automated threshold optimization of NDWI values enables preliminary water–land segmentation. To ensure cartographic accuracy, this process integrated an expert visual validation protocol that cross-verifies extracted water bodies against high-resolution basemaps. The segmentation threshold was iteratively refined until achieving > 95% classification accuracy (Cohen’s Kappa) through quantitative assessment of 500 randomly sampled coastal transects. Persistent false positives in landward regions underwent systematic removal via post-processing algorithms incorporating morphological refinement and contextual filtering, thereby generating aquaculture-specific masks with reduced commission errors (< 3%) for end-user applications.

(2) The Mask R-CNN model

Mask R-CNN [22,23,24] is an improved deep learning network for instance segmentation based on Faster R-CNN [25]. In this model, a standard convolutional neural network (CNN) and a feature pyramid network (FPN) were first used to extract features [26]. A region proposal network (RPN) was then used to find the target area. And finally, ROI Align was used to classify candidate areas and generate boundary boxes and masks. The loss function was set as the sum of the classification error, detection error, and segmentation error.

The Mask R-CNN model was trained in two stages. In the first stage, a part of the network was fixed to fully learn the parameters of the pre-training model. In the second stage, the whole network was liberalized and iterative training was carried out using stochastic gradient descent until the value of the loss function tended to be stable, and then the trained aquaculture area recognition model was obtained. The images to be recognized were input into the trained Mask R-CNN model to obtain the two extracted types of aquaculture area mask files, and the vector surface of the aquaculture area was obtained by raster to vector [27]. Using this vector, the area of aquaculture could be easily calculated. In order to improve the segmentation accuracy of the culture area, ResNet-101 was selected as the backbone network of the model [22].

(3) Model improvement with CBAM and Soft-NMS

To address the impact of complex background information in marine aquaculture areas on the model’s recognition accuracy and to improve the model’s precision in identifying elongated aquaculture zones, we optimized the model in two key aspects.

① The convolutional block attention module (CBAM) was integrated into the ResNet-101 feature extraction network to effectively suppress complex background noise and enhance the recognition accuracy of small targets, such as aquaculture areas [28].

② Mask R-CNN employed a region proposal network (RPN) to generate target region proposals, and the candidate regions were filtered using the non-maximum suppression (NMS) algorithm to eliminate redundant bounding boxes. However, the traditional NMS algorithm, which deletes candidate boxes with overlap ratios exceeding a predefined threshold, can inadvertently remove correct boxes, thereby negatively impacting the model’s performance. To address this issue, we introduced the Soft-NMS algorithm as an alternative to the conventional NMS. In Soft-NMS, the confidence penalty function can take two forms: linear and nonlinear [29]. The Gaussian function enables a more nuanced and gradual application of penalties. Specifically, as the overlap between boxes increases, the penalty on the confidence score initially rises slowly and then more sharply as the overlap becomes more significant. This nonlinear behavior more closely aligns with human perception, where a slight overlap may not significantly reduce confidence, but a substantial overlap leads to a marked decrease in confidence [30]. Additionally, the Gaussian penalty function is particularly effective in handling cases where multiple objects are closely spaced or partially occluded, as it differentiates penalties based on the degree of overlap. In contrast, a linear penalty function imposes a constant rate of confidence reduction regardless of the extent of overlap, which may not adequately preserve relevant detections [31]. Therefore, in this study, we opted for the nonlinear Gaussian penalty function, which imposes a greater penalty on the confidence score as the overlap increases, especially when the centers of the overlapping regions are closer. The formula used is as follows:

$$l_{i}=l_{i}e^{-\frac{iou{\left(M,b_{i}\right)}^{2}}{\sigma }},\forall b_{i}\notin D$$

where l*_i_* is the confidence, *M* is the candidate box with the highest score at present, *b_i_* is the pending box, and *D* is the final set of candidate boxes.

(4) Model combination

After obtaining the aquaculture area using the modified Mask R-CNN model, the topological relationship between the aquaculture area and the water area obtained by the NDWI could be determined so that land false detection could be eliminated and the detection result could be obtained.

### 2.3. Implementation Details

The initial learning rate was set to 0.001. The learning rate momentum was set to 0.9, and 10,000 iterations were performed. This model is packaged as a functional service module. By receiving this service through the client, users can adopt the model and identify aquaculture areas after uploading the images. The results include the location mark of aquaculture areas, the area counts of various types, the comparative analysis of the results in different periods, and the warning of the prohibited area, restricted area, and over-farming, allowing us to monitor the aquaculture areas.

## 3. Results and Discussion

### 3.1. Performance Analysis of the Improved Model

**Figure 3:**
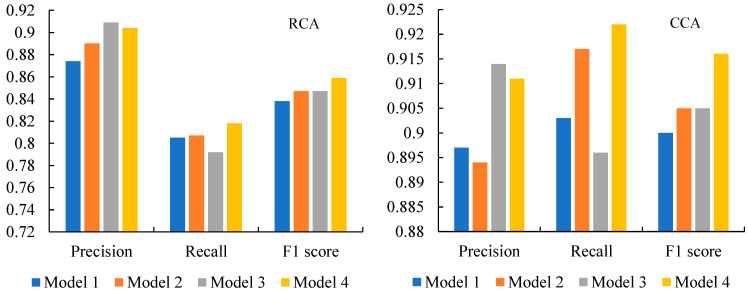
The performance of different models in the raft culture area (RCA) and the cage culture area (CCA). The basic Mask R-CNN model (referred to as “Model 1”). The Mask R-CNN model with the CBAM added (referred to as “Model 2”). The Mask R-CNN model with NMS replaced by Soft-NMS (referred to as “Model 3”). The Mask R-CNN model incorporating both the CBAM and Soft-NMS simultaneously (referred to as “Model 4”).

To evaluate the effectiveness of the two improvement strategies proposed in this study, ablation experiments were conducted using the Mask R-CNN model as a baseline. The experiments compared the impacts of incorporating the convolutional block attention module (CBAM) and replacing the traditional non-maximum suppression (NMS) with the Soft-NMS algorithm on the model’s performance. The high-resolution image dataset with a spatial resolution of 2 m from GF-1 was analyzed. Four different model configurations were tested, as shown in Figure 3.

As shown in Figure 3, the incorporation of the CBAM into Mask R-CNN effectively suppresses complex background information, resulting in a significant improvement in precision. Specifically, Model 2, which integrates the CBAM, achieves a 0.016 increase in precision compared to the baseline model, Model 1. However, the recall rate remains nearly unchanged, indicating that while the CBAM enhances the model’s ability to avoid false positives, it does not significantly boost the detection rate of true positives [32]. Overall, the F1 score of Model 2 is slightly higher than that of Model 1, reflecting a modest improvement in the model’s performance.

Replacing traditional NMS with Soft-NMS in Mask R-CNN further enhances the model’s precision. Model 3 demonstrates the highest precision among all models, with a 0.035 increase compared to the baseline. This improvement is attributed to Soft-NMS’s ability to avoid the over-removal of candidate boxes, a common issue with traditional NMS. However, the recall rate of Model 3 drops slightly, suggesting that Soft-NMS may be too aggressive in filtering out overlapping boxes, resulting in some true positives being missed. Despite this, the F1 score of Model 3 remains comparable to that of Model 2, indicating a balanced performance. Compared with the traditional Mask R-CNN model, the Mask R-CNN model incorporating both the CBAM and Soft-NMS has achieved certain improvements in precision, recall, and F1 score for both the RCA and the CCA. Specifically, the precision of the RCA has improved by about 3.4%, recall by about 1.6%, and F1 score by about 2.5%; for the CCA, the precision has improved by about 1.6%, recall by about 2.1%, and F1 score by about 1.8%. These improvements indicate that the introduction of the CBAM and Soft-NMS can effectively enhance the model’s performance, especially in balancing precision and recall.

The integration of the CBAM and Soft-NMS into Mask R-CNN significantly elevates its capacity for offshore aquaculture monitoring by addressing two critical challenges in marine remote sensing: background interference and overlapping target resolution. The CBAM enhances feature representation through its dual-channel attention mechanism: the channel attention submodule dynamically recalibrates spectral responses to suppress irrelevant coastal backgrounds (e.g., waves, ships, and algal blooms), while the spatial attention submodule sharpens the localization of elongated raft culture areas (RCAs) and densely packed cage culture areas (CCAs) by amplifying their unique geometric patterns [33]. Concurrently, Soft-NMS replaces traditional NMS with a decay function that preserves partially overlapping detections—a common scenario in aquaculture zones where CCA grids and RCA lines exhibit intricate spatial arrangements. This dual enhancement synergistically improves detection robustness: The CBAM reduces false positives of background noise in GF-1 imagery, while Soft-NMS increases recall for small/overlapping targets through adaptive confidence adjustment. These advancements highlight how attention-driven feature refinement and probabilistic suppression mechanisms jointly enable precise segmentation of aquaculture infrastructure, critical for sustainable coastal resource management.

### 3.2. Model Validation of Modified Mask R-CNN

**Figure 4:**
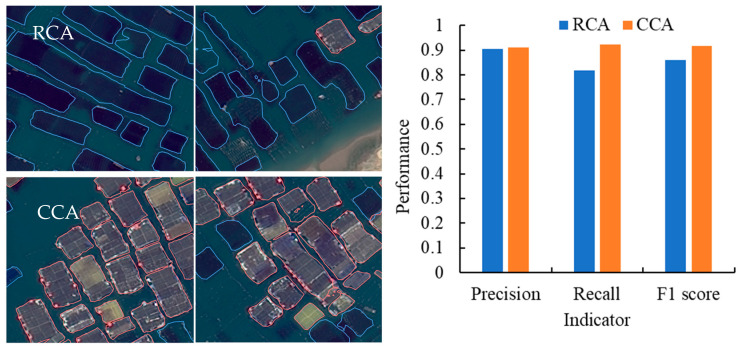
Model recognition effect and precision, recall, and F1 score of test samples in raft culture area (RCA, blue color) and cage culture area (CCA, red color).

The modified model training ended after 100 epochs, and then evaluation indicators were calculated quantitatively by using the test set, as shown in Figure 4. The precision, recall, and F1 score of the cage culture areas are 0.014, 0.069, and 0.043 higher than those of the raft culture areas, respectively, which may be related to the clearer boundaries of the cage culture areas.

Prior research documented precision, recall, and F1 scores for aquaculture area identification within the range of 0.79–0.98, 0.71–1.00, and 0.83–0.91, respectively [2]. The other research reported a precision of around 0.89 [34]. Our model significantly outperformed these benchmarks, achieving a precision of 0.904 for the RCA and 0.911 for the CCA, both exceeding the highest precision value (0.98) reported previously. The recall rates were 0.818 for the RCA and an impressive 0.922 for the CCA, nearing the maximum value (1.00) observed in prior studies. Our F1 scores were also robust, at 0.859 for the RCA and 0.916 for the CCA. This indicates that our model performs well in balancing precision and recall, particularly exhibiting high overall performance in identifying CCAs. In summary, our model has attained commendable precision, recall, and F1 scores, particularly excelling in the identification of CCAs. While the recall rate for RCAs could be further enhanced, the model has already proven itself to be highly reliable and practical, rendering it well suited for the task of identifying aquaculture areas against complex backgrounds.

### 3.3. Trend Analysis of Aquaculture Area Changes

**Figure 5:**
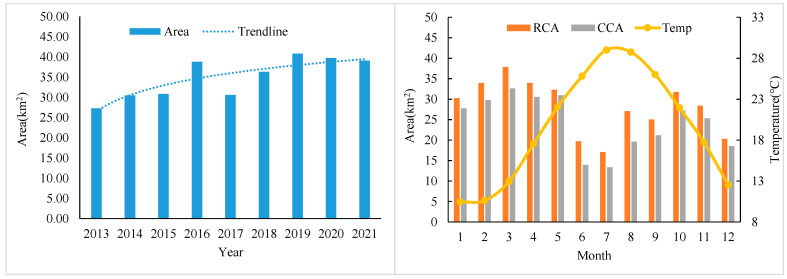
Average annual and monthly area of raft culture area (RCA) and cage culture area (CCA) at different air temperatures (Temp).

By identifying 36 images of the Xiapu area, the aquaculture area from 2013 to 2021 was obtained. Figure 5 shows the average annual and monthly aquaculture area in different years.

The aquaculture area in the Xiapu region has experienced significant changes over the years. From 2013 to 2021, the average annual aquaculture area initially increased, rising from 27.28 hectares in 2013 to a peak of 38.80 hectares in 2016. It then fluctuated between 30.58 hectares in 2017 and a high of 40.78 hectares in 2019, before stabilizing around 39 hectares in 2020 and 2021. The aquaculture areas show a periodic trend with distinct seasonal patterns. From January to May, the RCA and CCA both increase, peaking in March and April, respectively. This growth is likely due to favorable environmental conditions like rising water temperatures and longer daylight hours. However, a decline is observed in June, possibly due to summer monsoon rains and higher water temperatures. From June to August, the areas remain low, with the lowest point in July, corresponding to the hottest months. High temperatures and intense solar radiation can stress aquatic species and reduce productivity. From September to December, the RCA and CCA gradually increase again, with secondary peaks in October and November, respectively, as conditions improve and the monsoon season ends.

Xiapu is known for its diverse aquatic species, including but not limited to shellfish, fish, and seaweed. The temporal distribution of the aquaculture area observed in the data may reflect the specific growth cycles and environmental preferences of these species. It can be seen that their area changed with the seasons, which was closely related to the characteristics of local aquaculture behavior. In the Sandu’ao area, crops such as rhubarb fish, sea cucumber, abalone, kelp, and laver were mainly cultured. The raft culture areas with great changes in different periods in the images were mainly cultivated with kelp and laver. High temperature will cause the yield of these crops to decrease, so the best growing seasons are winter and spring. Kelp, the key cultivated crop in the Xiapu area, is cultivated around November and harvested from April to June of the following year. Laver is cultivated in September and harvested several times from October to February of the following year. Therefore, fewer raft culture areas were observed in June and July, and the total area of the aquaculture areas was at a minimum value.

Aquaculture areas are dynamic, and monitoring their changes helps reveal the characteristics of aquaculture behavior and contributes to managing the marine environment. However, in the study, which monitored and counted raft and cage culture in China’s offshore areas, the remote sensing data were based on single-view images from a representative year, reflecting only the aquaculture situation during a specific period [2]. Given the different climates and aquaculture practices in the north and south, this approach struggles to capture the actual aquaculture situation. The aquaculture area identification method used in this study, combining the NDWI and Mask R-CNN, addresses the issue of land misdetection and can effectively monitor changes in aquaculture areas. This method reflects changes in yield to some extent and is ultimately linked to seafood prices. Thus, dynamic monitoring of aquaculture areas aids in various aspects, including estimating aquaculture production, planning and optimizing aquaculture areas, and adjusting the aquaculture industry. This enables relevant authorities to better regulate aquaculture activities.

### 3.4. Violation Monitoring of Aquaculture Areas

**Figure 6:**
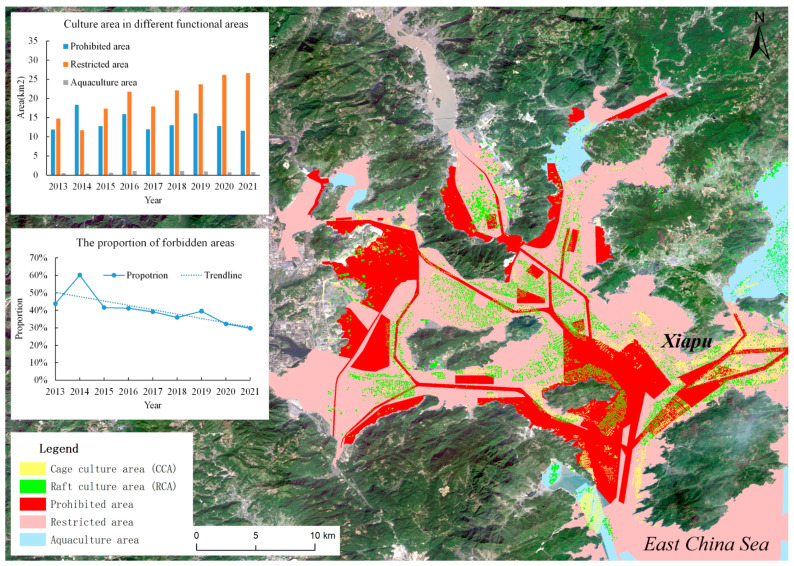
Location and quantity of raft culture area (RCA) and cage culture area (CCA) with functional areas in Xiapu.

As shown in Figure 6, the rapid growth in aquaculture in 2016 imposed considerable environmental pressure, prompting the local government to implement a series of measures in 2017, including marine water coating planning, which delineated prohibited and restricted aquaculture zones, among other regulations. Consequently, there was a notable decrease in the farming area in 2017. Nevertheless, due to the demands of economic development, the area began to increase substantially again after 2018–2019. This indicates that the implementation of aquaculture zone planning may not be effective, underscoring the necessity for continuous monitoring. To better monitor and manage aquaculture areas and to foster the sustainable development of aquaculture, this study conducted violation monitoring and warning analysis of aquaculture areas in conjunction with relevant government planning. The government put forward the planning scheme in 2018, which divided the aquaculture waters into three functional areas, which are the prohibited area, restricted area, and aquaculture area. Based on the scope of the culture area identified by the model, the culture area in the prohibited area was analyzed, and the results are shown in Figure 6.

Figure 6 reveals that numerous breeding areas are located within the designated prohibited breeding zones. Analysis of data since 2013 shows significant changes in the areas of prohibited, restricted, and aquaculture zones within the Xiapu marine aquaculture region, as well as variations in the proportion of prohibited areas. From 2013 to 2021, the prohibited area experienced fluctuations, starting at 11.94 km^2^ in 2013, peaking at 18.3533 km^2^ in 2014, then decreasing to 12.81 km^2^ in 2015, and rising again to 15.96 km^2^ in 2016. After the establishment of aquaculture functional zones in 2017, the prohibited area notably declined to 11.971 km^2^. However, it continued to oscillate, increasing to 13.04 km^2^ in 2018, 16.10 km^2^ in 2019, and then decreasing slightly to 12.80 km^2^ in 2020 and 11.61 km^2^ in 2021. In contrast, the restricted area consistently increased from 14.80 km^2^ in 2013 to 26.64 km^2^ in 2021. The aquaculture area also expanded, particularly after 2016, indicating an increase in aquaculture activities despite the presence of prohibited and restricted zones. This aligns with findings from some studies suggesting that the growth in China’s marine development activities has led to increased exploitation of marine resources and subsequent expansion of mariculture areas.

Regarding the proportion of prohibited areas, it was 44% in 2013, peaked at 60% in 2014, declined after 2017, reaching 39% in both 2017 and 2019, and further decreased to 32% in 2020 and 30% in 2021. This decline suggests that the establishment of aquaculture functional zones in 2017 had an impact on reducing the proportion of prohibited areas. Since 2013, there have been fluctuations in the areas of prohibited, restricted, and aquaculture zones. The establishment of aquaculture functional zones in 2017 appears to have contributed to a gradual decline in the proportion of prohibited areas. Relevant studies have found that since the establishment of marine aquaculture zones, the total number of these zones has decreased, yet the aquatic product output within these zones has been steadily increasing.

This is attributed to a significant improvement in the efficiency of marine aquaculture zones [35,36]. Although the proportion of the prohibited area has declined after the promulgation of the planning, it remains at a high level. Additionally, the proportion of the restricted area has increased significantly, necessitating a warning analysis for this area. Based on the actual situation of local aquaculture areas, specific warning standards should be formulated for the prohibited and restricted areas. By issuing warnings for areas that exceed these standards, the scientific planning of aquaculture by relevant departments can be effectively promoted, thereby achieving the green and sustainable development of mariculture.

## 4. Conclusions

To gain deeper insights into marine aquaculture practices, prevent regulatory oversight, and foster green and sustainable marine aquaculture development, this study introduces an aquaculture area recognition method leveraging the NDWI and an enhanced Mask R-CNN architecture with high-resolution remote sensing images. Compared with the traditional Mask R-CNN model, the Mask R-CNN model incorporating both the CBAM and Soft-NMS achieves a 3.4% increase in RCA precision and a 1.6% increase in CCA precision, demonstrating enhanced performance in balancing precision and recall. Based on this method, a monitoring framework for offshore aquaculture violations was established and applied to the Sandu’ao aquaculture area. By analyzing the Xiapu region as a case study, this study identified patterns of aquaculture area changes and critical monitoring periods aligned with local aquaculture activities. The results show a clear seasonal variation in aquaculture area, with a notable inverse correlation with temperature, underscoring the importance of monitoring during periods of significant change. Comparing planned prohibited and restricted areas with identified aquaculture areas during key periods revealed varying degrees of illegal aquaculture activities across different years, prompting a warning analysis of the aquaculture areas.

While the proposed method demonstrates efficacy in marine aquaculture monitoring, two limitations warrant consideration. The labor-intensive manual labeling process constrained dataset scalability, occasionally causing boundary ambiguities in low-resolution imagery that marginally affected area quantification accuracy. Additionally, although directly applicable to regions with analogous aquaculture practices and climates, implementation in ecologically dissimilar zones requires localized NDWI threshold calibration through field validation. Future research will prioritize practical measurement integration, including in situ validation campaigns using portable hyperspectral sensors to establish region-specific spectral thresholds, automated sensor networks to collect water quality parameters (e.g., dissolved oxygen, chlorophyll-a levels) at key aquaculture sites, and hybrid modeling frameworks that synergize satellite-derived boundaries with tidal gauge records and sediment load field data. These enhancements aim to strengthen detection robustness while maintaining operational feasibility for coastal management applications.

## Figures and Tables

**Table 1:** Details of the experimental data in different phases.

Research Phase	Source	Format	Time Range	Space Range	Spatial Resolution	Number of Images
Model training and validation	GF-1	.tif	13 June 2020	119°28′8″–120°9′44″ E, 26°21′34″–27°0′24″ N	2 m	1
Aquaculture area monitoring	Landsat-8	.tif	2013–2021	119°49′13″–119°57′3″ E, 26°36′38″–26°41′34″ N	15 m	36

**Table 2:** Characterization of dataset used for model training and validation.

Source	Spatial Resolution	Total Number of Image Samples	Training Samples	Validation Samples	Splitting Ratios
GF-1	2 m	80	64	16	4:1
Expanded GF-1	2 m	272	256	16	16:1
Bilinear down-sampling based on GF-1	4 m	272	256	16	16:1
	10 m	272	256	16	16:1
	15 m	272	256	16	16:1
	20 m	272	256	16	16:1
	30 m	272	256	16	16:1
	50 m	272	256	16	16:1
Landsat-8	15 m	2880	2160	720	4:1

## Data Availability

Restrictions apply to the availability of these data. Data were obtained from Chinese Academy for Environmental Planning and are available from the authors with the permission of Chinese Academy for Environmental Planning (http://www.caep.org.cn/).
